# NORAD-sponged miR-378c alleviates malignant behaviors of stomach adenocarcinoma via targeting NRP1

**DOI:** 10.1186/s12935-022-02474-5

**Published:** 2022-02-14

**Authors:** Yongjun Hu, Ming Luo

**Affiliations:** Department of General Surgery, The Second Xiangya Hospital, Central South University, No.139 Renmin Middle Road, Changsha, 410011 Hunan China

**Keywords:** Stomach adenocarcinoma, miR-378c, Lnc-NORAD, NRP1, EMT, Cell proliferation, Invasion, Tumor metastasis

## Abstract

**Background:**

Stomach adenocarcinoma (STAD) is the most common type of gastric cancer (GC), with a high recurrence rate and poor prognosis, but the potential indicators for STAD are insufficient.

**Methods:**

Herein, we found that MicroRNA-378c (miR-378c) was lowly expressed in STAD, and the low expression of miR-378c was highly correlated with poor overall survival (OS), T stage, Reflux history, DSS events and PFI events of STAD patients.

**Results:**

In addition, univariate analysis displayed that miR-378c was significantly associated with OS (Hazard ratio 0.735; 95% CI, 0.542–0.995; *P* = 0.046). Furthermore, it was validated that miR-378c inhibition accelerated STAD cell proliferation, migration, invasion and epithelial-mesenchymal transition (EMT), while they were suppressed by miR-378c overexpression. Mechanistically, Neuropilin 1 (NRP1) was confirmed as the target of miR-378c, and Lnc-NORAD was identified as its sponger. More importantly, NORAD-mediated miR-378c inhibited malignant behaviors of STAD both in vitro and in vivo.

**Conclusions:**

Collectively, these results suggest miR-378c as a promising indicator for the treatment of STAD.

**Supplementary Information:**

The online version contains supplementary material available at 10.1186/s12935-022-02474-5.

## Background

Stomach adenocarcinoma (STAD) is a type of gastric cancer (GC) [1], and caused by malignant transformation of gastric gland cells, accounting for 95% of gastric malignancies [2]. Histopathologically, gastric cancer can be classified into protruding type, penetrating type, disseminated type and mixed type [3]. Currently, radiotherapy [4], chemotherapy [5], tumor removal [6] and immunotherapy [7] are considered effective for gastric cancer. However, the high recurrence rate of gastric cancer is associated with the poor prognosis and high mortality. Therefore, determination of predictive biomarkers of STAD is extremely indispensable.

Long non-coding RNAs (LncRNAs) contain more than 200 nucleotides in length [8], and function as regulators in various biological behaviors, such as cell proliferation [9], migration [10], invasion [11] and EMT process [12]. LncRNAs like ABHD11-AS1 [9], TLCD2-1 [13] and HOTTIP [14] were highly correlated with progression of multiple cancers through activating or inhibiting gene expression. More specifically, Lu et al. ascertained that LncRNA NEAT1 upregulated ROCK1 through sponging miR-148b-3p to hinder retinoblastoma growth and metastasis [15]. Zuo et al. demonstrated that LINC01224 acted as a sponger of miR‑485‑5p to enhance AKT3 expression, thereby facilitating cell proliferation and inhibiting cell apoptosis in endometrial carcinoma. Additionally, Lnc-NORAD activated by oxidative stress sponged miR-433-3p and enhanced oxaliplatin resistance in gastric cancer through enhancing autophagy flux [16]. However, the contribution of NORAD to STAD remains unknown.

MicroRNAs, a type of small noncoding RNA molecules, are highly responsible for messenger mRNA expression [17]. Increasing evidence has unraveled the significance of MicroRNAs in gastric cancer. As Li et al. highlighted, Circ_0008035-sponged miR-599 restrained cell proliferation and induced its apoptosis and ferroptosis in GC through targeting EIF4A1, and Chen et al. disclosed that ASNR-targeted miR-519e-5p blocked tumor growth and metastasis via inhibiting FGFR2 in gastric cancer [12]. Although miR-378c has been identified as a potential biomarker for gastric cancer [18], the working mechanisms of miR-378c in STAD are unclear.

It was hypothesized that there was a ceRNA mechanism between NORAD and miR-378c, so we detected the expression of miR-378c and NORAD in STAD, and validated their relationship. In addition, the functional role of miR-378c in vitro and in vivo was confirmed. Overall, our paper may provide novel insights for the therapy of STAD.

## Methods

### Prediction of potential targets and lncRNAs for miR-378c

The genes highly expressed in STAD were downloaded from the GEPIA database [19] and intersected with the top 500 survival genes in this database and the target genes predicted by starBase [20] for miR-378c. The overlapped genes were potential target genes for miR-378c in STAD. Highly expressed lncRNAs in STAD were extracted from TCGA database and intersected with lncRNAs predicted to adsorb miR-378c by starBase. The overlapped genes were potential lncRNAs adsorbing miR-378c in STAD.

### Cell culture and transfection

Stomach adenocarcinoma cell lines MGC-803 and MKN-28, and Human gastric mucosal cells GES-1 were purchased from the Type Culture Collection of the Chinese Academy of Sciences (Shanghai, China), and were incubated at 37 °C in DMEM (Gibco, Thermo Fisher Scientific, Inc., Jiangsu, China) with 10% FBS (cat. no. 12483020, Gibco, Thermo Fisher Scientific, Inc., Jiangsu, China).

The pcDNA-NORAD, pcDNA-NRP1, miR-378c-mimics, and miR-378c-inhibitors were obtained from Genomeditech (Shanghai, China), and NORAD or NRP1 shRNA was inserted into the lentiviral vector pLKO.1 (Genechem, Shanghai, China) to knock down NORAD or NRP1. Then, they were transfected into MGC-803 and MKN-28 cells based on the protocols of lipofectamine (11668-019, Invitrogen, USA).

### Colony formation

To determine cell proliferation, cells were seeded into 6-well plates at a density of 1000 cells per well and cultured for 2 weeks. Then 4% paraformaldehyde was added for fixation and 0.1% crystal violet was added for dye. Finally, the cloned cells were observed and calculated.

### BrdU staining

BrdU incorporation assay was performed for the estimation of MGC-803 and MKN-28 cell proliferation. In brief, MGC-803 and MKN-28 cells were incubated in coverslips, followed by mixing with BrdU (20 μM) for 4 h. Then, PBS containing 0.1% Triton X-100 was added to permeabilize these cells and 3% FBS solution was used for blocking. DNasel treatment was used to denature cellular DNA. The Alexa Fluor 647 anti-BrdU monoclonal antibody (BD Biosciences, USA) was added for incorporated BrdU staining, and a Carl Zeiss fluorescence microscope was used for visualization.

### Transwell assay

Transwell chambers (BD Bioscience, US) were used for determination of MGC-803 and MKN-28 cell migration and invasion. In brief, 100 μL serum-free DMEM containing a total of 10^5^ MGC-803 or MKN-28 cells was added into the upper chambers pre-coated with Matrigel (BD Bioscience). Meanwhile, complete medium was added into the lower chambers to induce cell invasion. After 24-h incubation, cells in the lower surface were fixed by paraformaldehyde, and stained by 0.1% crystal violet. The results were observed under a microscope (Olympus) at least 6 fields of view.

### Wound healing assay

To monitor the migration of MGC-803 and MKN-28 cells, a 200-µL sterile pipette tip was used to generate the scratch, and the treated cells were incubated on DMEM containing 10% FBS. At 0 and 24 h, the wound area was quantified using ImageJ software version 1.8.0–112 (National Institutes of Health, MA).

### Luciferase reporter assay

To quantify the luciferase activity, miR-378c mimics and NC-mimics were co-transfected with NRP1-MUT or NRP1-WT for 24 h, and the LncNORAD-MUT or LncNORAD-WT was transfected into HEK293T cells. The dual-luciferase reporter assay system (Promega Corp, Madison, US) was used to quantify the luciferase activity.

### Xenograft and lung metastasis models and HE staining

To construct xenograft and lung metastasis models, we first constructed miR-378c-agomir + NRP1, miR-378c-agomir + LncNORAD, miR-378c-agomir and miR-378c-agomir + Vector MGC-803 cells, and these cells were respectively injected into the flank or tail vein of BALB/c nude mice (female, 6 weeks old). 4 weeks after injection, these nude mice were sacrificed after intravenous injection of barbiturate at a final concentration of 100 mg/kg and the xenografts tumor and lung metastatic nodules were collected. The volume of xenograft tumor was monitored using the formula below: volume (mm^3^) = (L × W2)/2 (L: length, W: width), and the tumor weight and number of lung metastatic foci were measured. The collected lung metastatic tissues were fixed, dehydrated and embedded using 4% paraformaldehyde, ethanol and wax, respectively. Based on the manufacturer manual, HE staining was performed, and the tissues were observed histologically.

### Immunohistochemistry (IHC) assay

In brief, the paraffin embedded xenograft tumor tissues were boiled in citrate buffer for antigen retrieval, and the permeabilization was performed using PBS which contained 0.2% Triton-X. Subsequently, the treated tissues were incubated at 4 °C overnight with antibodies of Ki67 (ab92742, 1:500) and cleaved caspase-3 (ab32042, 1:500), and then cultured with secondary antibody. 3,3′-diaminobenzidine substrate (DAB) staining was performed, and Ki67-positive cells in tissues were observed under a light microscope.

### RNA immunoprecipitation (RIP)

EZ-Magna RIP kit (Merck KGaA) was used to determine the relationship of miR-378c with NRP1 and LncNORAD. In brief, after washed with PBS, MGC-803 cells were lysed by RIP lysis buffer, and then the lysates were exposed to the magnetic beads-coated AGO2 antibody or IgG antibody. Then the total RNA was extracted after the RNA complex was eluted from magnetic beads, and the expressions of LncNORAD and NRP1 were detected using qRT-PCR.

### Western blotting assay

MGC-803 and MKN-28 cells were lysed by RIPA lysis buffer, and the collected lysates were added with protease inhibitor PMSF. Then BCA method was used for the determination of protein concentrations, and SDS-PAGE electrophoresis was performed to separate protein samples. Next, the isolated samples were transferred onto skim milk powder blocked PVDF membranes for 1 h, and then mixed with these membranes in a hybridization box overnight at 4 °C, followed by incubation with secondary antibody for 1 h. The bands were visualized using ECL luminescent solution. The primary antibodies NRP1 (ab81321,1/2000), NRP2 (ab273584,1/1000), E-cadherin (ab76055,1:200), N-cadherin (ab76011,1:5000), Vimentin (ab92547,1:2000), bcl-2 (ab32124,1:1000), bax (ab32503,1:2000), cleaved caspase-3 (ab32042,1:500), caspase-3 (ab32351, 1:5000), cleaved caspase-9 (ab2324, 1 µg/ml), and caspase-9 (ab32539,1:1000) were used.

### qRT-PCR

For RNA quantification, TRIzol reagent (Invitrogen, Carlsbad, California) was utilized for the extraction of total RNA, and the cDNA was obtained after RNA was reversely transcribed using PrimeScript RT Reagent kit (Invitrogen, Carlsbad, California), while mirPremier microRNA Isolation Kit (Sigma, St. Louis, Missouri) was used for miRNA extraction. SYBR Prime Script RT-PCR kit (Takara, Dalian, China) was used for the quantitative real-time PCR based on the ABI 7300 rapid real-time PCR system (Applied Biosystems, Foster City, California). For results calculation, the 2^−ΔΔCt^ formula was adopted, and the primer sequences are displayed in Additional file 1: Table S1.

### Statistical analysis

In our work, SPSS version 20. 0 (SPSS Inc., Chicago, Illinois) was utilized for statistical analysis and all data were presented as mean ± SEM. Kaplan–Meier method was employed for the overall survival assessment, and differences between two groups or multiple groups were respectively analyzed by Student’s t-test and analysis of variance (ANOVA). *P* < 0.05 indicated significant difference.

## Results

### miR-378c is lowly expressed in STAD

**Fig. 1:**
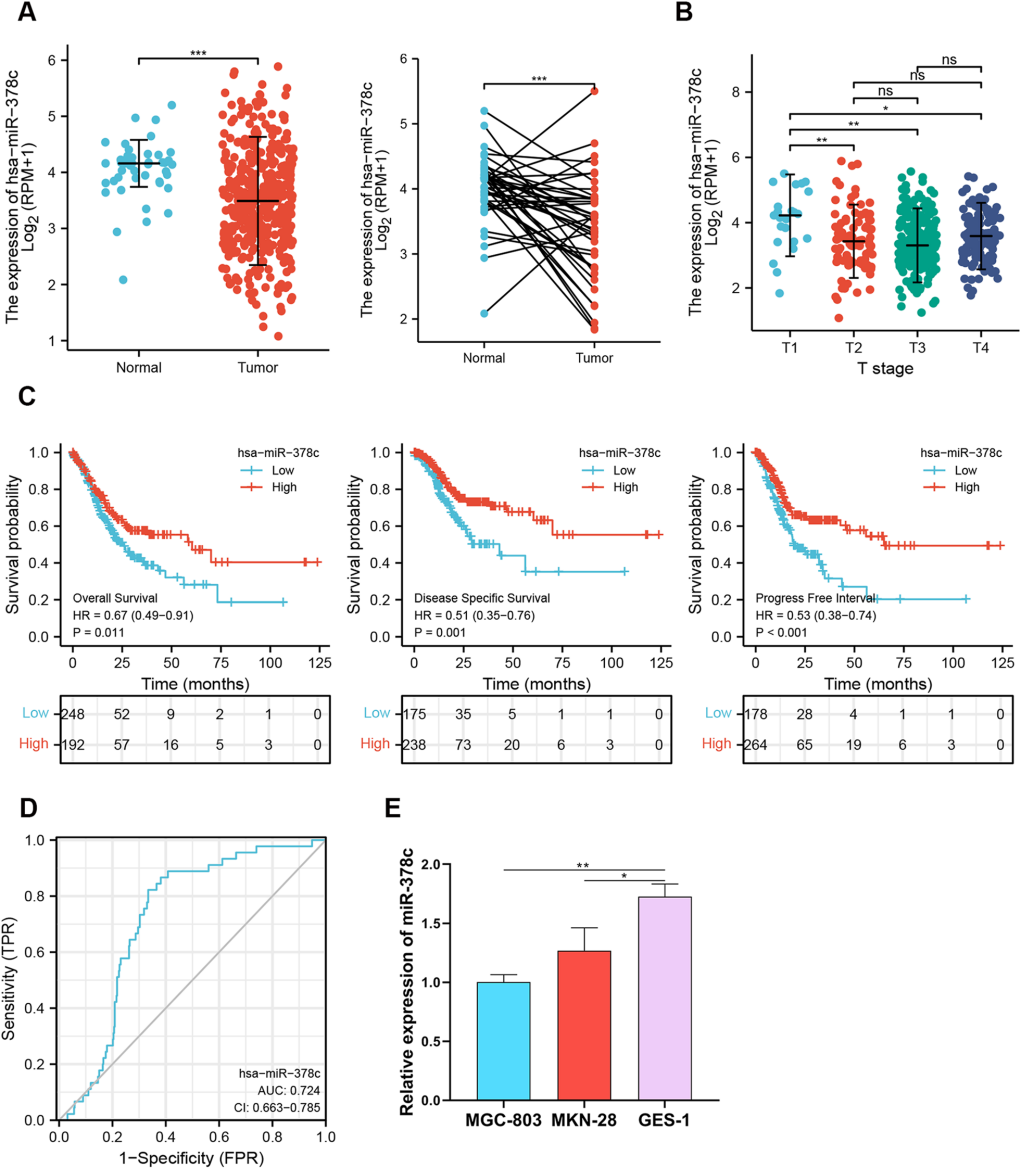
miR-378c is lowly expressed in STAD. miR-378c expression in STAD in TCGA database (**A**, **B**). The survival analysis (**C**) and ROC curve (**D**) of STAD. qRT-PCR was used to detect miR-378c expression in STAD cell lines MGC-803 and MKN-28, and human gastric mucosal cells GES-1 (**E**). *P < 0.05, **P < 0.01, ***P < 0.001. Data represent at least three independent sets of experiment

miR-378c has been considered as a potential biomarker for gastric cancer [18], while its functional contribution to STAD remains elusive. Herein, we first analyzed miR-378c expression in STAD based on TCGA database, and it was concluded that miR-378c was lowly expressed in STAD tumor (Fig. 1A), and was downregulated at T2-T4 stages compared with T1 stage (Fig. 1B). In addition, the low expression of miR-378c was highly correlated with the poor overall survival (Fig. 1C), T stage, Reflux history, DSS events and PFI events (Table 1) of STAD patients. Univariate analysis indicated that the expression of miR-378c was correlated with T stage (T3 and T4 and T2 vs. T1), N stage (N1 and N2 and N3 vs. N0), M stage (M1 vs. M0), pathologic stage (Stage II and Stage III and Stage IV vs. Stage I), age (> 65 vs. <  = 65), and residual tumor (R1 and R2 vs. R0), while multivariate analysis displayed that the expression of miR-378c was closely correlated with N stage, age and residual tumor (Table 2). Furthermore, the ROC curve indicated a high AUC value of miR-378c in STAD, suggesting that miR-378c may be considered as a biomarker of STAD (Fig. 1D). To further confirm miR-378c expression in STAD, q-PCR demonstrated that miR-378c was lowly expressed in STAD cell lines MGC-803 and MKN-28, in comparison with human gastric mucosal cells GES-1 (Fig. 1E). Collectively, these results suggested that miR-378c was inhibited in STAD, and its low expression indicated poor prognosis of STAD patients.

**Table 1:** Correlation between hsa-miR-378c expression and clinicopathologic characteristics of gastric cancer patients

Characteristic	Low expression of hsa-miR-378c	High expression of hsa-miR-378c	p
n	223	223	
T stage, n (%)			0.009
T1	6 (1.4%)	17 (3.9%)	
T2	51 (11.7%)	41 (9.4%)	
T3	111 (25.5%)	88 (20.2%)	
T4	52 (11.9%)	70 (16.1%)	
N stage, n (%)			0.699
N0	60 (14.1%)	71 (16.7%)	
N1	62 (14.6%)	57 (13.4%)	
N2	44 (10.3%)	43 (10.1%)	
N3	47 (11%)	42 (9.9%)	
M stage, n (%)			0.570
M0	195 (46%)	199 (46.9%)	
M1	17 (4%)	13 (3.1%)	
Pathologic stage, n (%)			0.665
Stage I	25 (6%)	33 (7.9%)	
Stage II	69 (16.5%)	62 (14.8%)	
Stage III	96 (22.9%)	91 (21.7%)	
Stage IV	22 (5.3%)	21 (5%)	
Primary therapy outcome, n (%)			0.337
PD	42 (11.2%)	31 (8.3%)	
SD	10 (2.7%)	8 (2.1%)	
PR	5 (1.3%)	2 (0.5%)	
CR	133 (35.5%)	144 (38.4%)	
Gender, n (%)			0.197
Female	71 (15.9%)	85 (19.1%)	
Male	152 (34.1%)	138 (30.9%)	
Race, n (%)			0.662
Asian	50 (13%)	43 (11.2%)	
Black or African American	6 (1.6%)	7 (1.8%)	
White	135 (35.2%)	143 (37.2%)	
Histological type, n (%)			0.851
Diffuse Type	36 (8.1%)	36 (8.1%)	
Mucinous Type	13 (2.9%)	9 (2%)	
Not Otherwise Specified	122 (27.5%)	126 (28.4%)	
Papillary Type	6 (1.4%)	3 (0.7%)	
Signet Ring Type	6 (1.4%)	6 (1.4%)	
Tubular Type	38 (8.6%)	42 (9.5%)	
Residual tumor, n (%)			0.450
R0	173 (44.6%)	178 (45.9%)	
R1	12 (3.1%)	7 (1.8%)	
R2	10 (2.6%)	8 (2.1%)	
Histologic grade, n (%)			0.569
G1	6 (1.4%)	4 (0.9%)	
G2	75 (17.2%)	85 (19.5%)	
G3	137 (31.4%)	130 (29.7%)	
Anatomic neoplasm subdivision, n (%)			0.239
Antrum/Distal	80 (18.7%)	83 (19.4%)	
Cardia/Proximal	39 (9.1%)	25 (5.8%)	
Fundus/Body	76 (17.8%)	75 (17.5%)	
Gastroesophageal Junction	19 (4.4%)	27 (6.3%)	
Other	1 (0.2%)	3 (0.7%)	
Reflux history, n (%)			0.022
No	115 (44.7%)	94 (36.6%)	
Yes	17 (6.6%)	31 (12.1%)	
Antireflux treatment, n (%)			0.307
No	94 (43.5%)	79 (36.6%)	
Yes	19 (8.8%)	24 (11.1%)	
H pylori infection, n (%)			0.089
No	79 (42.7%)	86 (46.5%)	
Yes	5 (2.7%)	15 (8.1%)	
Barretts esophagus, n (%)			1.000
No	110 (44.2%)	121 (48.6%)	
Yes	9 (3.6%)	9 (3.6%)	
OS event, n (%)			0.120
Alive	128 (28.7%)	145 (32.5%)	
Dead	95 (21.3%)	78 (17.5%)	
DSS event, n (%)			0.021
Alive	149 (35.6%)	168 (40.1%)	
Dead	62 (14.8%)	40 (9.5%)	
PFI event, n (%)			0.020
Alive	139 (31.2%)	163 (36.5%)	
Dead	84 (18.8%)	60 (13.5%)	
Age, meidan (IQR)	66 (58, 72)	68 (58, 74)	0.231

**Table 2:** Univariate and multivariate Cox regression analyses of clinical characteristics associated with overall survival

Characteristics	Total(N)	Univariate analysis		Multivariate analysis	
		Hazard ratio (95% CI)	P value	Hazard ratio (95% CI)	P value
T stage (T3 and T4 and T2 vs. T1)	430	10.412 (1.458–74.379)	**0.020**	5.421 (0.685–42.884)	0.109
N stage (N1 and N2 and N3 vs. N0)	420	2.190 (1.474–3.253)	** < 0.001**	2.088 (1.173–3.718)	**0.012**
M stage (M1 vs. M0)	420	2.571 (1.553–4.258)	** < 0.001**	1.842 (0.992–3.420)	0.053
Pathologic stage (Stage II and Stage III and Stage IV vs. Stage I)	413	2.303 (1.276–4.157)	**0.006**	0.936 (0.389–2.251)	0.883
Gender (Male vs. Female)	440	1.026 (0.747–1.409)	0.875		
Race (Black or African American and White vs. Asian)	381	1.380 (0.894–2.129)	0.146		
Age (> 65 vs. < = 65)	437	1.472 (1.080–2.005)	**0.014**	1.570 (1.089–2.264)	**0.016**
Histologic grade (G2 and G3 vs. G1)	431	1.001 (0.319–3.138)	0.999		
Residual tumor (R1 and R2 vs. R0)	383	3.064 (1.953–4.806)	** < 0.001**	2.006 (1.186–3.392)	**0.009**
Reflux history (Yes vs. No)	255	0.612 (0.326–1.148)	0.126		
Antireflux treatment (Yes vs. No)	215	0.705 (0.404–1.231)	0.219		
H pylori infection (Yes vs. No)	184	0.483 (0.194–1.202)	0.118		
Barretts esophagus (Yes vs. No)	247	0.869 (0.353–2.141)	0.761		
hsa-miR-378c (High vs. Low)	440	0.735 (0.542–0.995)	**0.046**	0.803 (0.566–1.140)	0.220

### miR-378c suppresses cell proliferation, invasion and EMT of STAD

**Fig. 2:**
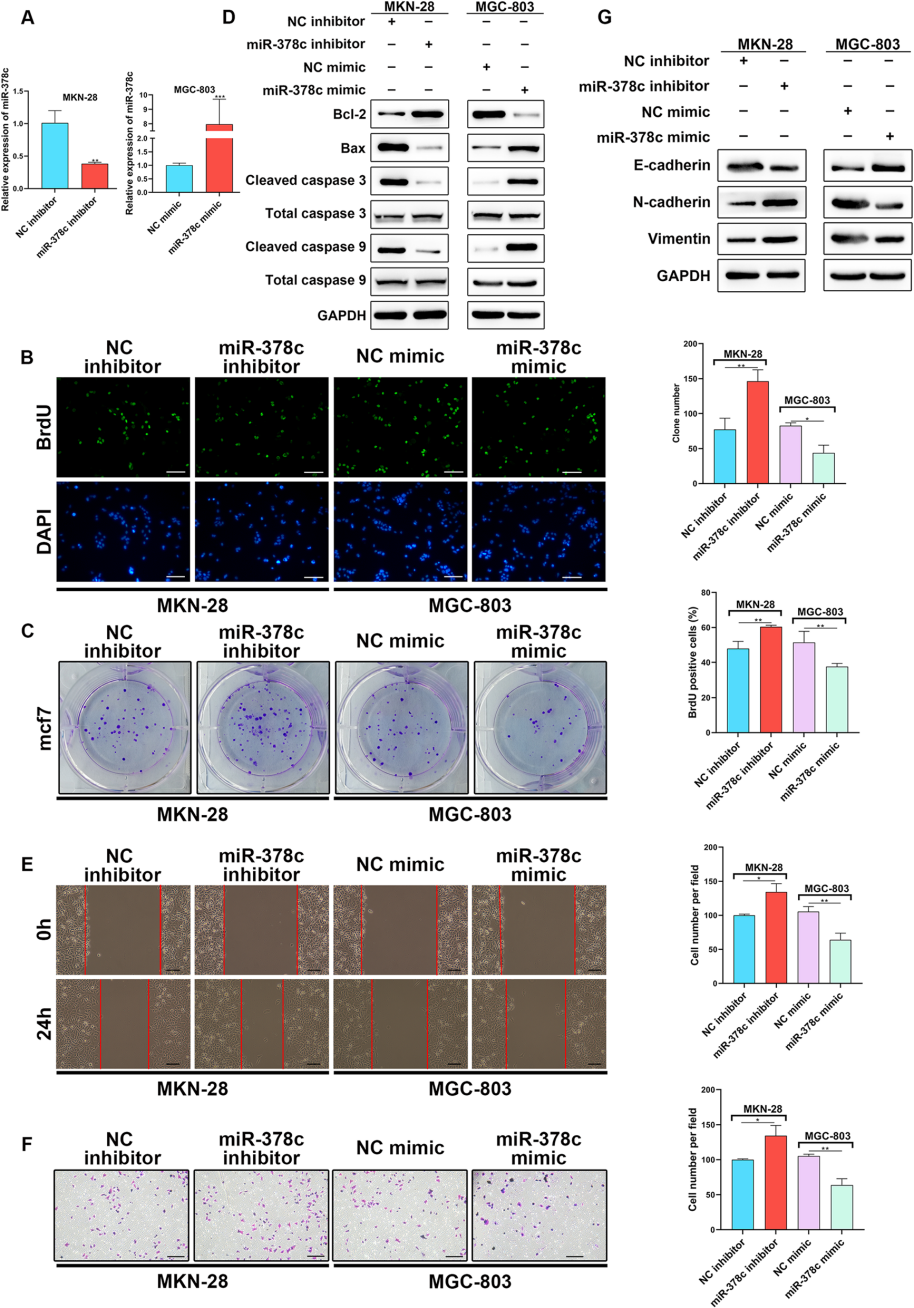
miR-378c suppresses cell proliferation, invasion and EMT of STAD. qRT-PCR was used to examine the overexpression or down-regulation of miR-378c in MGC-803 or MKN-28 cells (**A**). STAD cell proliferation was determined by BrdU staining (**B**, scale bar, 100 μm) and colony formation (**C**, scale bar, 1 cm) assays. Apoptosis-related proteins were determined by Western blotting (**D**). Wound healing (**E**, scale bar, 100 μm) and Transwell (**F**, scale bar, 100 μm) assays validated the migratory and invasive capacities of STAD cells. The EMT-related proteins were determined by Western blotting (**G**). *P < 0.05, **P < 0.01, ***P < 0.001. Data represent at least three independent sets of experiment

To investigate the functional role of miR-378c in STAD, we constructed miR-378c-inhibted MKN-28 cells and miR-378c-overexpressed MGC-803 cells (Fig. 2A). BrdU staining (Fig. 2B) and colony formation (Fig. 2C) assays indicated that miR-378c depletion enhanced MKN-28 cell viability, while it was repressed by miR-378c overexpression. For determination of apoptosis-related proteins (Fig. 2D), Western blotting demonstrated that depleted miR-378c induced the upregulation of Bcl-2, and the downregulation of Bax, Cleaved capase 3 and Cleaved capase 9, but it was reversed by overexpressed miR-378c. Furthermore, wound healing (Fig. 2E) and Transwell (Fig. 2F) assays validated that the migratory and invasive capacities of STAD cells were enhanced by miR-378c silencing, while they were weakened by miR-378c upregulation. Additionally, it was found that miR-378c overexpression blocked the EMT process of STAD, which was specifically indicated by the increased expression of E-cadherin and the reduced expression of Vimentin and N-cadherin (Fig. 2G). Overall, these findings confirmed the inhibitory role of miR-378c in biological behaviors of STAD cells.

### miR-378c inhibits NRP1 in STAD

**Fig. 3:**
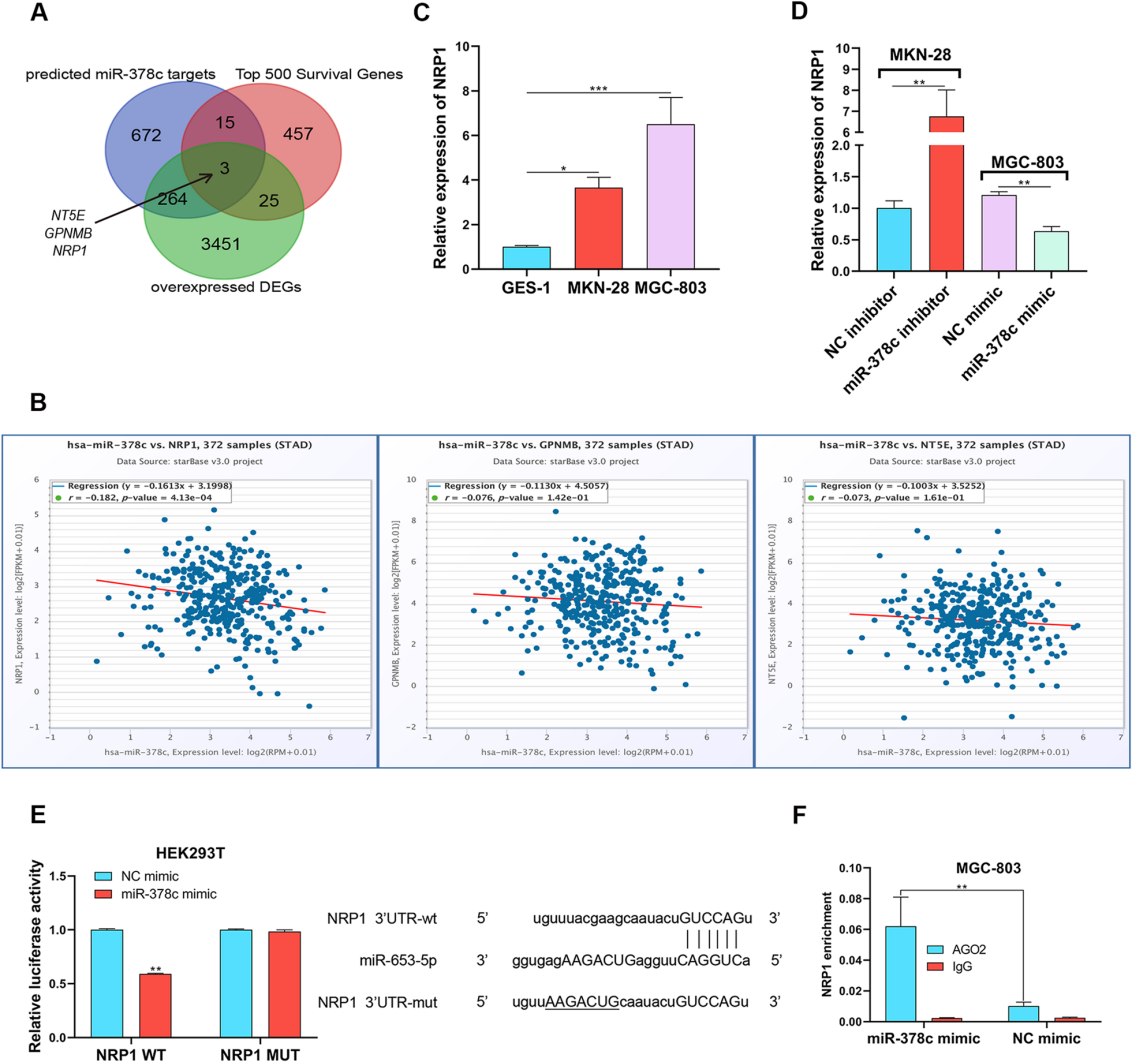
miR-378c inhibits NRP1 in STAD. Screened genes from Starbase and GEPIA database were intersected with Top 500 Survival Genes to obtain the targets of miR-378c (**A**). The correlation analysis between miR-378c and NRP1 (**B**). NRP1 expression in gastric cancer cells (**C**), miR-378c-depleted MKN-28 cells, and miR-378c-overexpressed MGC-803 cells was determined by qRT − PCR (**D**). Dual Luciferase reporter (**E**) and RIP (**F**) assays were performed to determine the relationship between miR-378c and NRP1. **P < 0.01. Data represent at least three independent sets of experiment

To disclose the target of miR-378c in STAD, we intersected the Starbase predicted miR-378c targets, the Top 500 Survival Genes and the differentially upregulated genes from GEPIA database, and screened NT5E, GPNMB and NRP1 (Fig. 3A). According to correlation analyses, it was found that there was a negative correlation between miR-378c and NRP1 (Fig. 3B). Then, we detected the NRP1 expression in GES-1 cell line and gastric cancer cell lines. We found that NRP1 expression was highest in MGC-803 cells (Fig. 3C) and was enhanced by miR-378c depletion while suppressed by miR-378c upregulation (Fig. 3D). Moreover, it was observed that miR-378c overexpression obviously reduced luciferase activity of NRP1-WT, but there were no changes in NRP1-WT group (Fig. 3E). In addition, RIP assay further verified that miR-378c mimics strongly enriched NRP1 in AGO2 antibodies rather than in IgG, compared with NC mimics (Fig. 3F). In summary, our results validated the negative correlation between miR-378c and NRP1 in STAD.

### miR-378c hinders malignant behaviors of STAD cells through targeting NRP1

**Fig. 4:**
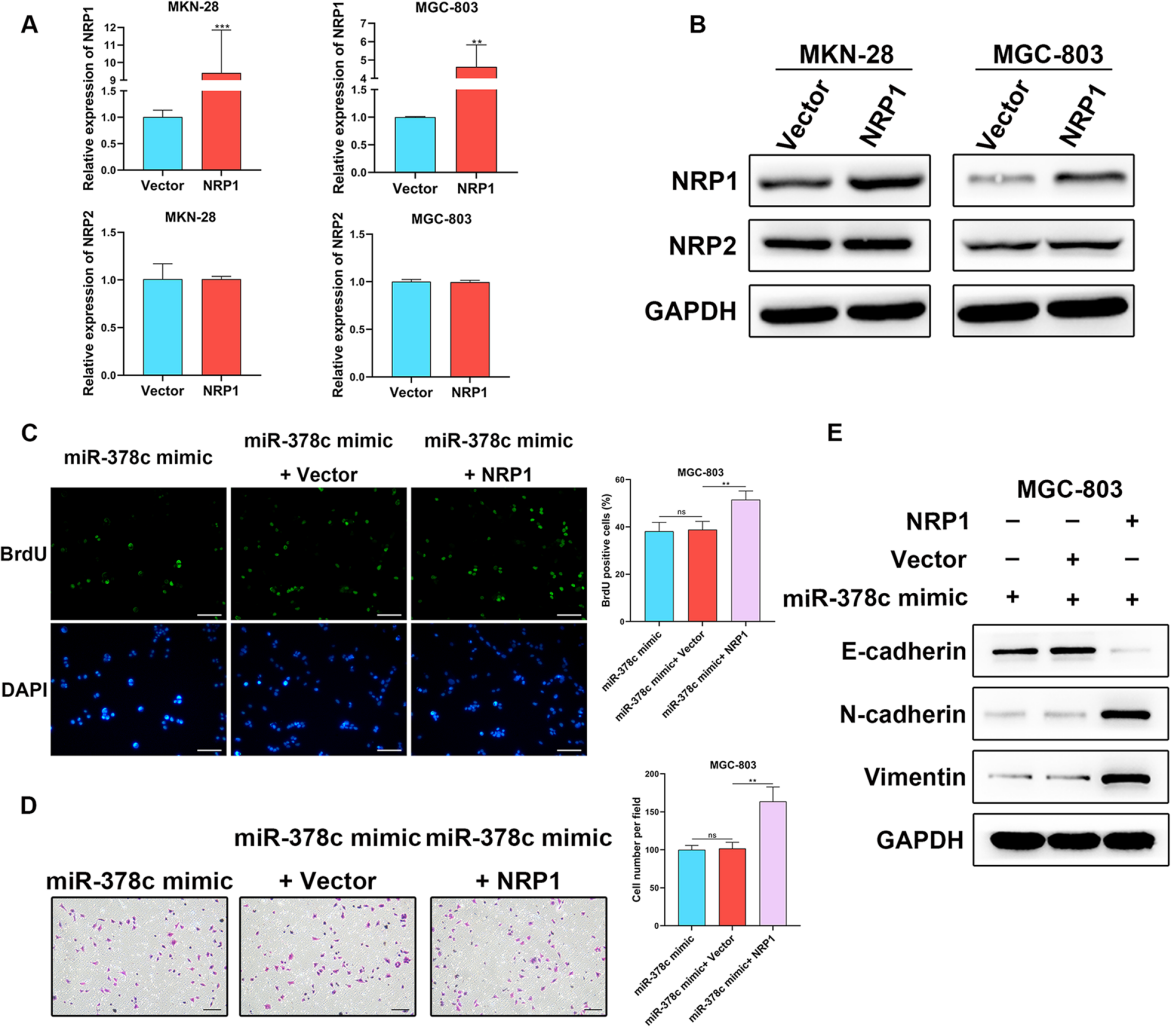
miR-378c hinders malignant behaviors of STAD cells through targeting NRP1. qRT−PCR (**A**) and Western blotting (**B**) were performed to determine NRP1 and NRP2 expressions in NRP1-overexpressed MGC-803 and MKN-28 cells. BrdU staining (**C**, scale bar, 100 μm) and Transwell (**D**, scale bar, 100 μm) assay were respectively applied to evaluate cell proliferation and invasion. The EMT-related proteins were determined by Western blotting (**E**). **P < 0.01, ***P < 0.001. Data represent at least three independent sets of experiment

To further determine the function of NRP1 in STAD cells, we constructed NRP1-overexpressed MGC-803 and MKN-28 cells, and the results showed that pcDNA-NRP1 promoted NRP1 expression and didn’t affect the expression of NRP2, an important paralog of NRP1, which indicated that NRP1 was successfully overexpressed (Fig. 4A, B). After miR-378c mimics and NRP1 plasmids were con-transfected into MGC-803 cells, BrdU staining assay demonstrated that miR-378c overexpression-induced impairment of cell proliferation was rescued by NRP1 overexpression (Fig. 4C). Furthermore, upregulated NRP1 also acted as a rescuer in remedying miR-378c overexpression-induced loss of invasive capacity of MGC-803 cells (Fig. 4D). In addition, it was found that miR-378c mimics enhanced expression of E-cadherin, which was restrained by NRP1 overexpression (Fig. 4E). Collectively, our results indicated that miR-378c suppressed malignant behaviors of STAD cells via inhibiting NRP1.

### miR-378c silence promotes malignant behaviors of STAD cells via NRP1

**Fig. 5:**
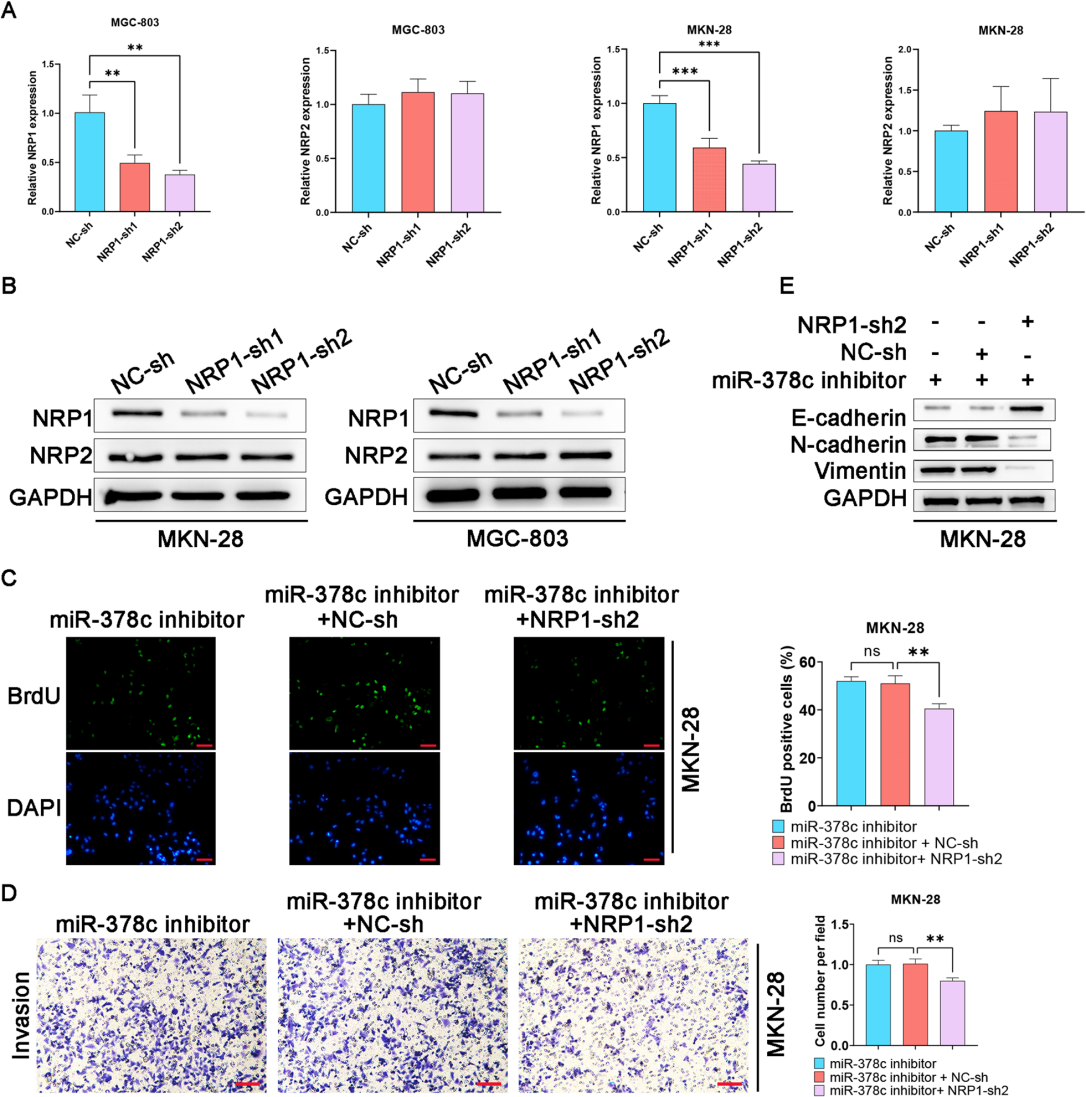
miR-378c inhibitor promotes malignant behaviors of STAD cells via NRP1. qRT-PCR (**A**) and Western blotting (**B**) were performed to determine NRP1 and NRP2 expressions in NRP1-silenced MGC-803 and MKN-28 cells. BrdU staining (**C**, scale bar, 100 μm) and Transwell (**D**, scale bar, 100 μm) assay were respectively applied to evaluate cell proliferation and invasion. The EMT-related proteins were determined by Western blotting (**E**). **P < 0.01, ***P < 0.001. Data represent at least three independent sets of experiment

To further determine whether NRP1 could rescue the effects of miR-378c silence in STAD cells, we first constructed NRP1-knocked down MGC-803 and MKN-28 cells, and it was found that the mRNA and protein expression of NRP1 was downregulated, while NRP2 was not affected (Fig. 5A, B). After miR-378c inhibitor and NRP1-sh were con-transfected into MKN-28 cells, BrdU staining assay demonstrated that miR-378c inhibitor-induced cell proliferation was rescued by NRP1 silence (Fig. 5C). Furthermore, inhibition of NRP1 also reduced miR-378c inhibitor-induced invasion of MGC-803 cells (Fig. 5D). In addition, it was found that miR-378c inhibitor enhanced expression of N-cadherin and Vimentin, which was restrained by NRP1 inhibition (Fig. 5E). Collectively, our results indicated that NRP1 inhibition could partly reverse the effects of miR-378c inhibitor on malignant behaviors of STAD cells.

### miR-378c is sponged by NORAD in STAD cells

**Fig. 6:**
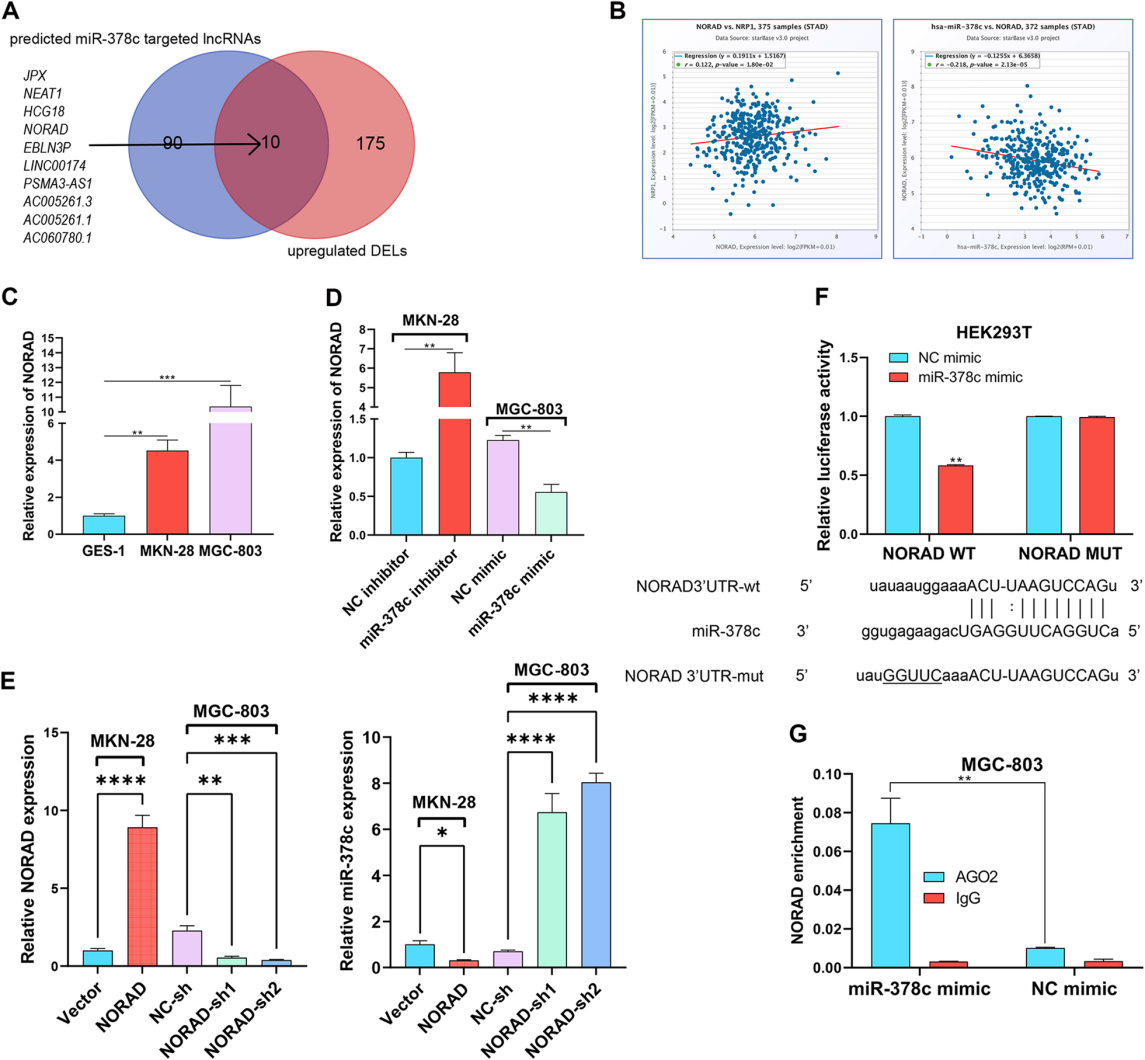
miR-378c is sponged by NORAD in STAD cells. Starbase and GEO database were adopted to identify miR-378c targeted LncRNAs (**A**). The correlation analysis between miR-378c and NORAD (**B**). NORAD expression in gastric cancer cells (**C**), miR-378c-depleted MKN-28 cells, and miR-378c-overexpressed MGC-803 cells was determined by qRT-PCR (**D**). miR-378c and NORAD expression in NORAD-depleted MKN-28 cells, and NORAD-overexpressed MGC-803 cells was determined by qRT-PCR (**E**). Dual Luciferase reporter (**F**) and RIP (**G**) assays were performed to determine the relationship between miR-378c and NORAD. **P < 0.01. Data represent at least three independent sets of experiment

As we know, ceRNA underlies cancer development, including STAD [21]. For the investigation of the upstream regulator of miR-378c, we performed intersection using Starbase and predicted miR-378c targeted LncRNAs and differentially upregulated LncRNAs in STAD. As a result, JPX, NEAT1, HCG18, NORAD, EBLN3P, LINC00174, PSMA3-AS1, AC005261.3, AC005261.1 and AC060780.1 were identified (Fig. 6A). Among them, it was found that NORAD was negatively correlated with miR-378c and positively correlated with NRP1 (Fig. 6B). In addition, NORAD expression was highest in MGC-803 cells (Fig. 6C) and was enhanced by miR-378c depletion, while suppressed by miR-378c overexpression (Fig. 6D). Besides, miR-378c expression could be inhibited by NORAD overexpression and promoted by NORAD silence (Fig. 6E). For further confirmation, Dual luciferase reporter assay was performed, and it was found that miR-378c mimics noticeably reduced the luciferase activity of NORAD WT, while NORAD MUT was not affected (Fig. 6F). Additionally, it was obviously observed that miR-378c mimics abounded NORAD in anti-AGO2 complexes, rather than in IgG antibody (Fig. 6G). Taken together, these data illustrated that NORAD functioned as a sponger of miR-378c in STAD cells.

### NORAD drives malignant behaviors of STAD cells via sponging miR-378c

**Fig. 7:**
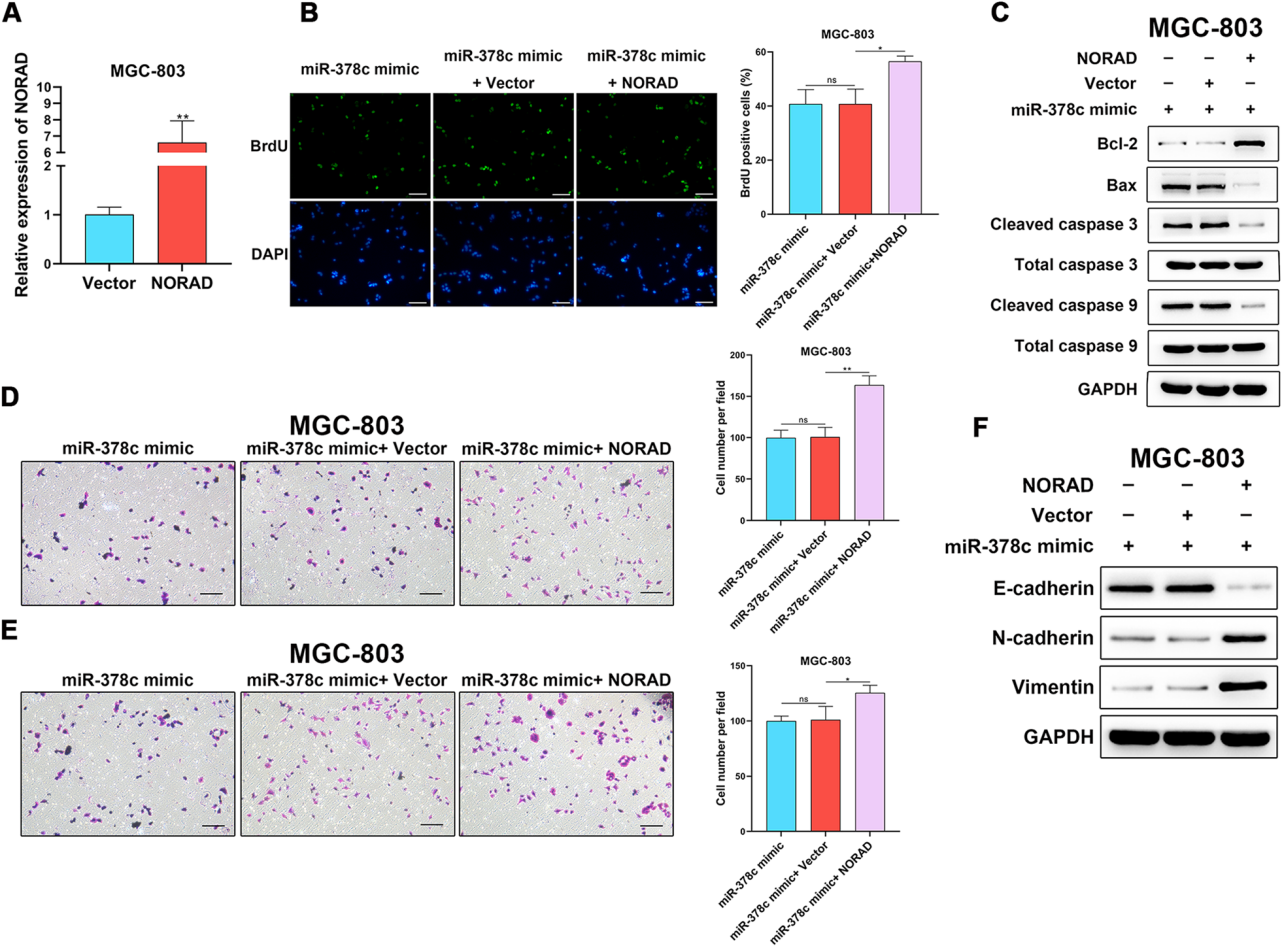
NORAD drives malignant behaviors of STAD cells via sponging miR-378c. qRT-PCR was utilized to examine the overexpression efficiency of NORAD in MGC-803 cells (**A**). BrdU staining was performed to measure cell proliferation (**B**, scale bar, 100 μm), and apoptosis-related proteins were determined by Western blotting (**C**). Transwell assay was performed to determine MGC-803 cell migration (**D**, scale bar, 100 μm) and invasion (**E**, scale bar, 100 μm), and the EMT-related proteins were determined by Western blotting (**F**). *P < 0.05, **P < 0.01. Data represent at least three independent sets of experiment

To unravel whether miR-378c mediates biological function of NORAD in STAD cells, we first detected the overexpression efficiency of NORAD in MGC-803 cells (Fig. 7A). Next, it was found that overexpressed NORAD rescued miR-378c overexpression-induced loss of STAD cell viability (Fig. 7B). In addition, as an anti-apoptotic protein, Bcl-2 was significantly inhibited by miR-378c overexpression, while rescued by upregulated NORAD (Fig. 7C). Consistently, miR-378c-alleviated migration and invasion of STAD cells were remedied by NORAD (Fig. 7D, E). Moreover, overexpressed NORAD accelerated EMT process, and inhibited expression of E-cadherin, and increased expression of N-cadherin and Vimentin were observed (Fig. 7F). In summary, NORAD induced malignant behaviors of STAD cells via mediating miR-378c.

### NORAD silence inhibits malignant behaviors of STAD cells via promoting miR-378c

**Fig. 8:**
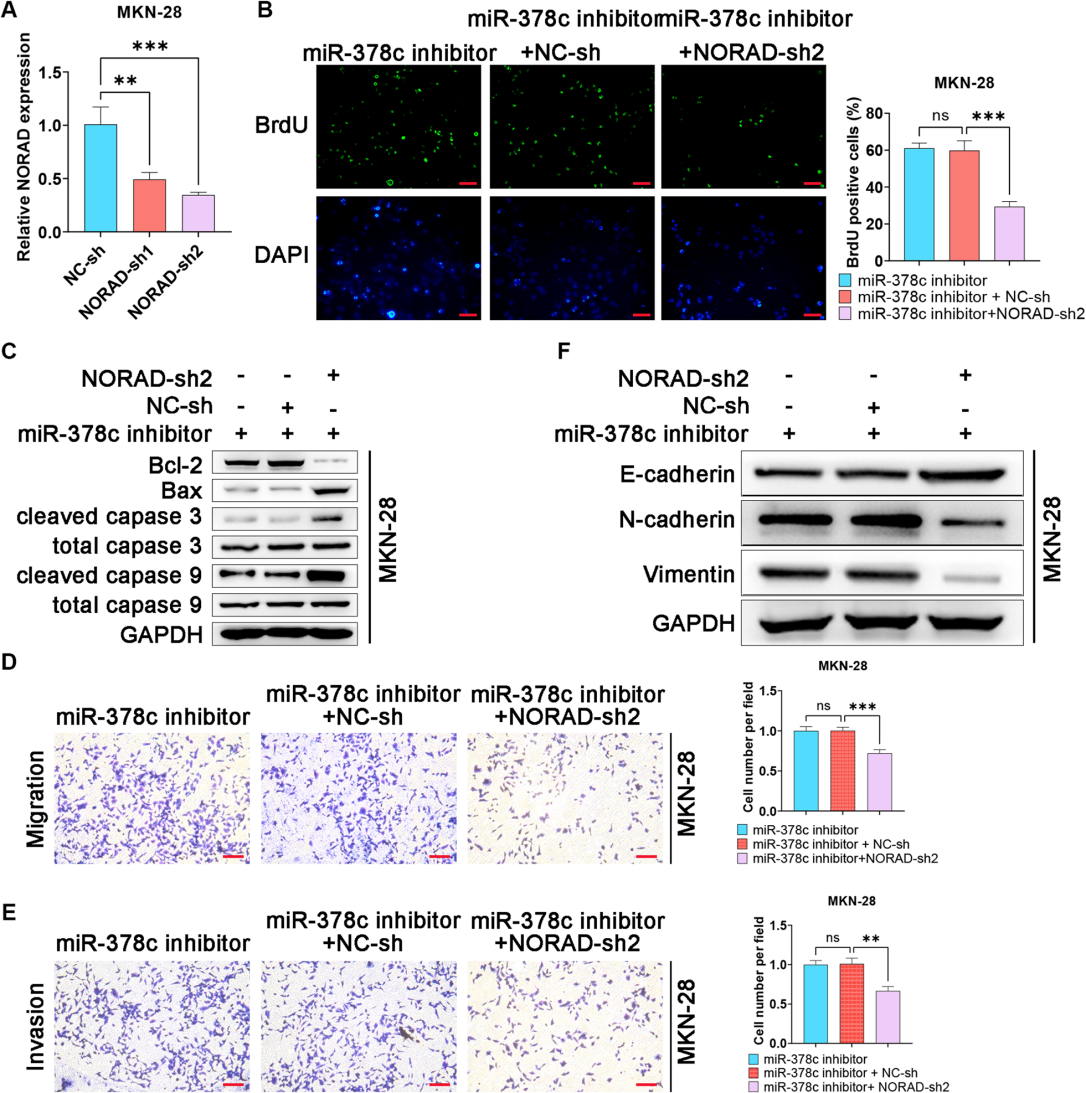
NORAD silence inhibits malignant behaviors of STAD cells via promoting miR-378c. qRT-PCR was utilized to examine the silence efficiency of NORAD in MKN-28 cells (**A**). BrdU staining was performed to measure cell proliferation (**B**, scale bar, 100 μm), and apoptosis-related proteins were determined by Western blotting (**C**). Transwell assay was performed to determine MGC-803 cell migration (**D**, scale bar, 100 μm) and invasion (**E**, scale bar, 100 μm). EMT-related proteins were determined by Western blotting (**F**). *P < 0.05, **P < 0.01. Data represent at least three independent sets of experiment

Next, we detected the silence efficiency of NORAD in MKN-28 cells (Fig. 8A). BrdU assay showed that NORAD knock down could rescue the miR-378c inhibitor-induced STAD cell proliferation (Fig. 8B). In addition, as an anti-apoptotic protein, Bcl-2 was significantly enhanced by miR-378c inhibitor, while rescued by downregulated NORAD (Fig. 8C). Consistently, miR-378c inhibitor-induced migration and invasion of STAD cells were alleviated by NORAD (Fig. 8D, E). Moreover, NORAD silence inhibited miR-378c inhibitor-induced EMT process (Fig. 8F). In summary, NORAD silence inhibited malignant behaviors of STAD cells by promoting miR-378c.

### miR-378c alleviates STAD tumor growth and lung metastasis in vivo through NORAD/NRP1 axis

**Fig. 9:**
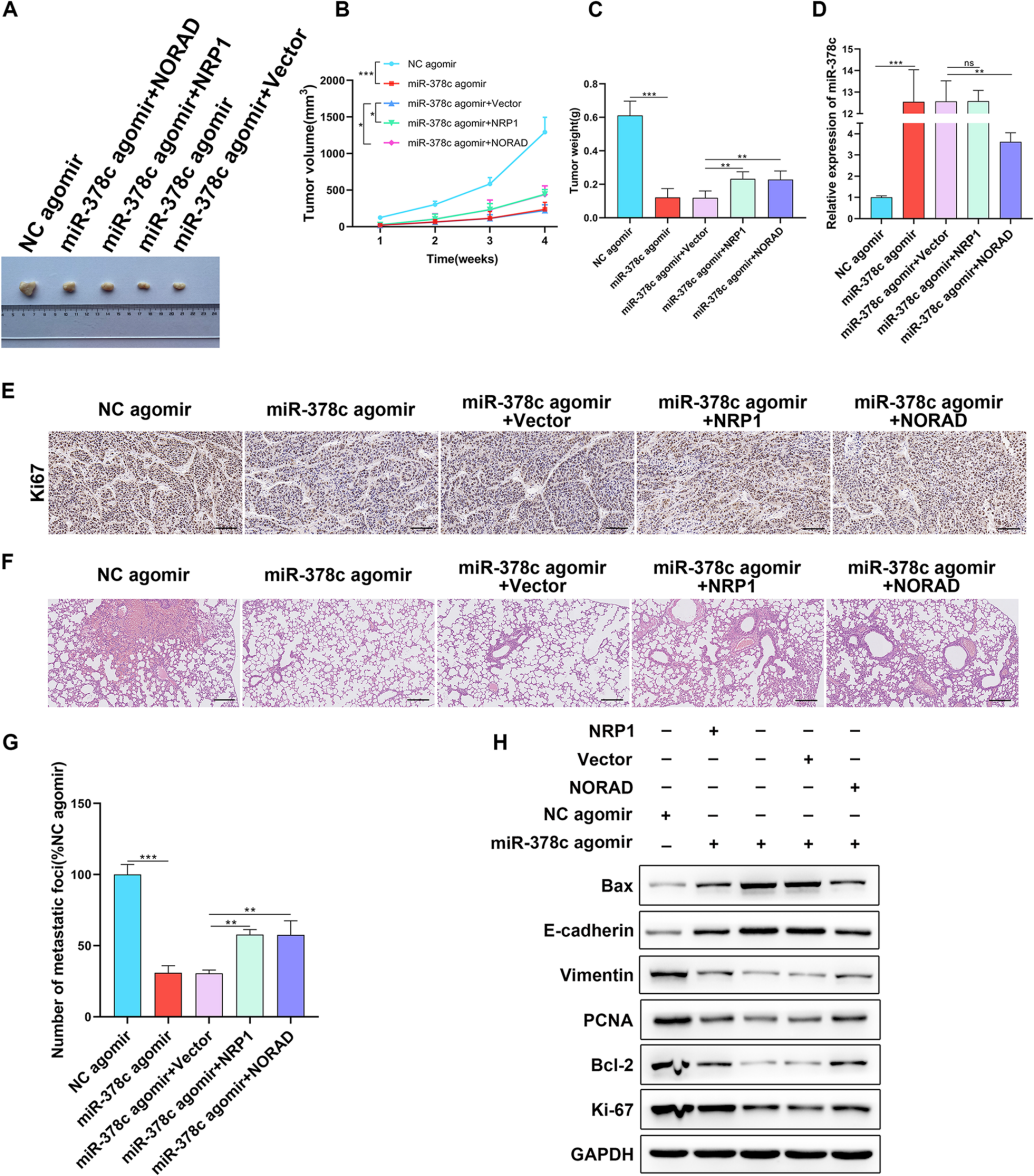
miR-378c alleviates STAD tumor growth and lung metastasis in vivo through NORAD/NRP1 axis. Representative tumor images (**A**). Tumor volume (**B**) and weight (**C**) were monitored. qRT-PCR was utilized to examine miR-378c expression in tumor tissues (**D**). IHC staining were performed to detect Ki67 expression and cleaved caspase 3 expression in tumor tissues (**E**). HE staining was performed to estimate the histopathology of tumor metastasis (**F**, scale bar, 100 μm), and the number of metastatic foci was counted (**G**, scale bar, 100 μm). Western blotting was used to measure the proliferation, apoptosis, and EMT related proteins (**H**). **P < 0.01, ***P < 0.001. Data represent at least three independent sets of experiment

To validate whether miR-378c inhibits STAD progression in vivo through modulating NORAD/NRP1 axis, miR-378c and NRP1 overexpressed or miR-378c and NORAD overexpressed MGC-803 cells were co-injected into the flank or tail vein of nude mice to construct xenograft and lung metastasis models. Tumor growth was monitored, and it was found that miR-378c-agomir-induced loss of tumor volume (Fig. 9A, B) and weight (Fig. 9C) was partly rescued by NRP1 or NORAD overexpression. Moreover, miR-378c expression in the tumor was examined by q-PCR (Fig. 9D). In addition, IHC staining displayed reduction of Ki67 and cleaved caspase 3 in the xenograft tumor with miR-378c-overexpressed MGC-803 cells. However, they were reversed by co-injection of NRP1 or NORAD overexpression cells (Fig. 9E). HE staining showed that compared with NC-agomir group, miR-378c-agomir alleviated the metastasis and infiltrative growth of metastatic tumors, which was rescued by injecting NRP1 or NORAD-overexpressed cells (Fig. 9F). Meanwhile, NRP1 or NORAD upregulation also increased the number of metastatic foci (Fig. 9G). Notably, miR-378c suppressed proliferation and EMT process and promoted apoptosis in vivo, while it was rescued by NRP1 or NORAD overexpression (Fig. 9H). Overall, it was validated that miR-378c inhibited tumor growth and lung metastasis of STAD in vivo through mediating NORAD/NRP1 axis.

## Discussion

STAD is the most common malignant tumor of gastric cancer, and is characterized by a high incidence. Up to date, the scarcity of biomarkers for the diagnosis of STAD is responsible for the poor overall survival of STAD patients. Recently, the indispensable role of miRNAs in STAD has been revealed. Liu et al. demonstrated that transcription factor STAT4-activated miR-3619-5p accelerated carcinogenesis and progression of stomach adenocarcinoma through targeting TBC1D10B [22]. In contrast, Xing et al. highlighted that miR-4521 acted as an inhibitor in regulating STAD tumor metastasis and EMT via inhibiting IGF2 and FOXM1 [23], and Ye et al. suggested that inhibited miR-7 facilitated STAD progression, and was related to the poor clinical outcomes [24]. In our work, we first revealed downregulation of miR-378c in STAD tissues based on bioinformatics analysis, and it was found that the low expression of miR-378c was closely associated with the poor prognosis, T stage, reflux history, DSS events and PFI events of STAD patients. Interestingly, univariate analysis exhibited that miR-378c was significantly correlated with OS (Hazard ratio 0.735; 95% CI, 0.542–0.995; *P* = 0.046), and the ROC curve displayed that miR-378c was of high diagnostic significance in STAD, with AUC = 0.724. These bioinformatics results indicated that miR-378c was closely associated with the clinical progression of STAD and may participate in STAD development. According to the functional experiments, it was found that upregulated miR-378c could repress STAD cell proliferation, migration, invasion and EMT metastasis, while depleted miR-378c facilitated these biological behaviors, which further validated the inhibitory role of miR-378c in the malignant behaviors of STAD.

As previous evidence indicated, miRNAs function through binding to the 3'UTR of targets [17]. Herein, NRP1 was identified as the target of miR-378c based on Dual luciferase reporter and RIP assays, and it was upregulated in STAD. Notably, through rescue experiments, it was validated that miR-378c inhibited malignant behaviors of STAD cells in a NRP1-dependent manner. Consistent with our research, Kang et al. revealed the upregulation of NRP1 in STAD, and it was demonstrated that its high expression indicated poor overall survival [25], and Wang et al. validated the close association between NRP1 and malignant phenotypes of STAD patients [26].

LncRNAs like MIR503HG [27], LINC01224 [28] and HAND2-AS1 [29] have been proved to be involved in the regulation of malignant phenotypes in STAD. Specifically, Li et al. found BRD4-mediated LncRNA MAGI2-AS3 maintained the upregulation of ZEB1 to accelerate malignant progression of STAD through sponging miR-141/200a [30], and Li et al. elaborated that ELF3-mediated lncRNA UBE2CP3 stabilized ITGA2 expression as a sponger of miR-138-5p to drive gastric cancer metastasis [31]. Similarly, Lnc-NORAD in our research was verified as the sponger of miR-378c, and accelerated malignant behaviors through inhibiting miR-378c. More importantly, xenograft and lung metastasis models were constructed, and it was confirmed that NORAD-sponged miR-378c blocked STAD tumor growth and metastasis in vivo through inhibiting miR-378c, which was supported by the loss of tumor weight, volume and metastatic number.

## Conclusions

In conclusion, our results clarified the downregulation of miR-378c in STAD, and identified NRP1 as the target and NORAD as the sponger of miR-378c. Moreover, it was validated that NORAD-mediated miR-378c inhibited malignant behaviors of STAD both in vitro and in vivo, which may be regarded as potential candidates against STAD. Small molecule drugs targeting NORAD and miR-378c may be beneficial in the treatment of STAD.

## Supplementary Information

**Additional file 1.** **Table S1.** Sequences of PCR primers used in this study.

## Data Availability

Not applicable.

## Abbreviations

STAD: Stomach adenocarcinoma
GC: Gastric cancer
miR-378c: MicroRNA-378c
OS: Overall survival
EMT: Epithelial–mesenchymal transition
NRP1: Neuropilin 1
LncRNAs: Long non-coding RNAs
IHC: Immunohistochemistry
RIP: RNA immunoprecipitation
