# Supplementary material for: NORAD-sponged miR-378c alleviates malignant behaviors of stomach adenocarcinoma via targeting NRP1
Source: Cancer Cell Int. 2022 Feb 14;22:79. doi: 10.1186/s12935-022-02474-5 (PMC8842946; doi:10.1186/s12935-022-02474-5)
Supplement: Supplementary file 1 — Additional file 1. Table S1. Sequences of PCR primers used in this study. [file 12935_2022_2474_MOESM1_ESM.docx]

**Table S1. Sequences of PCR primers used in this study**

| miR-378c | Forward(5’-3’) | GCGCACTGGACTTGGAGTC |
| --- | --- | --- |
|  | Reverse(5’-3’) | CAGTGCGTGTCGTGGAGT |
| GAPDH | Forward(5’-3’) | TGTGGGCATCAATGGATTTGG |
|  | Reverse(5’-3’) | ACACCATGTATTCCGGGTCAAT |
| NORAD | Forward(5’-3’) | TGATAGGATACATCTTGGACATGGA |
|  | Reverse(5’-3’) | AACCTAATGAACAAGTCCTGACATACA |
| NRP1 | Forward(5’-3’) | GGCGCTTTTCGCAACGATAAA |
|  | Reverse(5’-3’) | TCGCATTTTTCACTTGGGTGAT |
| NRP2 | Forward(5’-3’) | GCTGGCTACATCACTTCCCC |
|  | Reverse(5’-3’) | GGGCGTAGACAATCCACTCA |
